# Supplementary material for: Oncometabolite fumarate impairs ATR-CHK1 signaling by succinating RPA1 in Fumarate Hydratase-deficient renal cell carcinoma cells
Source: bioRxiv. 2025 Dec 10:2025.12.08.692638. Preprint. [Version 1] doi: 10.64898/2025.12.08.692638 (PMC12713586; doi:10.64898/2025.12.08.692638)
Supplement: Supplement 1 — Supplementary Table-S1: Top 30 PSMs among 930 succinated proteins identified by MS in UOK268 cells Supplementary Table-S2: Succinated 25 ssDNA binding proteins identified by MS in UOK268 cells [file media-1.pdf]

Supplementary Table S1: Top 30 PSMs among 930 succinated proteins identified by MS in UOK268 cells

Accession	GenSymbol	Protein names	AAs*	Summed PSM**
Q09666	AHNK	Neuroblast differentiation-associated protein AHNAK	5890	400
P35580	MYH10	Myosin-10 (Cellular myosin heavy chain, type B)	1976	198
P07437	TBB5	Tubulin beta chain (Tubulin beta-5 chain)	444	170
P68371	TBB4B	Tubulin beta-4B chain (Tubulin beta-2 chain)	445	154
P35579	MYH9	Myosin-9 (Cellular myosin heavy chain, type A)	1960	147
Q13885	TBB2A	Tubulin beta-2A chain (Tubulin beta class IIa)	445	138
Q9BVA1	TBB2B	Tubulin beta-2B chain	445	136
Q9BQE3	TBA1C	Tubulin alpha-1C chain (Alpha-tubulin 6)	449	128
Q71U36	TBA1A	Tubulin alpha-1A chain (Alpha-tubulin 3)	451	135
P04350	TBB4A	Tubulin beta-4A chain (Tubulin 5 beta) (Tubulin beta-4 chain)	444	126
Q02413	DSG1	Desmoglein-1 (Cadherin family member 4)	1049	121
Q07954	LRP1	Prolow-density lipoprotein receptor-related protein 1 (LRP-1)	4544	112
P52272	HNRPM	Heterogeneous nuclear ribonucleoprotein M (hnRNP M)	730	106
P0DOX5	IGG1	Immunoglobulin gamma-1 heavy chain	449	107
Q9UQE7	SMC3	Structural maintenance of chromosomes protein	1217	91
P19338	NUCL	Nucleolin (Protein C23)	710	90
P60709	ACTB	Actin, cytoplasmic 1 (Beta-actin)	375	98
P07355	ANXA2	Annexin A2 (Annexin II) (Annexin-2) (Calpactin I heavy chain)	339	80
P0DMV9	HS71B	Heat shock 70 kDa protein 1B (Heat shock 70 kDa protein 2)	641	76
Q08211	DHX9	ATP-dependent RNA helicase A (EC 3.6.4.13	1270	75
P06748	NPM	Nucleophosmin (NPM) (Nucleolar phosphoprotein B23)	294	74
Q14683	SMC1A	Structural maintenance of chromosomes protein 1A	1233	74
Q9BUF5	TBB6	Tubulin beta-6 chain (Tubulin beta class V)	446	73
Q13835	PKP1	Plakophilin-1 (Band 6 protein) (B6P)	747	66
Q14562	DHX8	ATP-dependent RNA helicase DHX8 (EC 3.6.4.13)	1220	64
P09874	PARP1	Poly [ADP-ribose] polymerase 1 (PARP-1) (EC 2.4.2.30)	1014	64
P23246	SFPQ	Splicing factor, proline- and glutamine-rich	707	64
P01859	IGHG2	Immunoglobulin heavy constant gamma 2	326	110
Q15233	NONO	Non-POU domain-containing octamer-binding protein	471	59
P05388	RLA0	60S acidic ribosomal protein P0 (60S ribosomal protein L10E)	317	59

***AAs : amino acid numbers; **PSM : peptide-spectrum matches**

Supplementary Table -S2 :Succinated 25 ssDNA binding proteins identified by MS in UOK268 cells

Gene name	Protein name	PSMs
DHX9	DEAH box protein 9	75
HNRNPA2B1	Heterogeneous nuclear ribonucleoproteins A2/B1	43
HNRNPA1	Heterogeneous nuclear ribonucleoprotein A1	42
HNRNPU	Heterogeneous nuclear ribonucleoprotein U	35
SMC2	Structural maintenance of chromosomes protein 2	14
SMC4	Structural maintenance of chromosomes protein 4	10
POLR2H	DNA-directed RNA polymerases I, II, and III subunit RPABC3	7
MCM7	DNA replication licensing factor MCM7	7
YBX1	Y-box-binding protein 1	6
HNRNPDL	Heterogeneous nuclear ribonucleoprotein D-like	6
SAMHD1	Deoxynucleoside triphosphate triphosphohydrolase	5
SUB1	Activated RNA polymerase II transcriptional coactivator p15	4
RPA1	Replication protein A 70 kDa DNA-binding subunit	4
HSPD1	60 kDa heat shock protein, mitochondrial	4
PCBP1	Poly(rC)-binding protein 1	4
PCBP2	Poly(rC)-binding protein	4
NME1	Nucleoside diphosphate kinase A	4
SSBP1	Single-stranded DNA-binding protein, mitochondrial	4
POLR2G	DNA-directed RNA polymerase II subunit RPB7	3
PURA	Purine-rich single-stranded DNA-binding protein alpha	3
TOP1	DNA topoisomerase 1	3
PURB	Purine-rich element-binding protein B	2
MCM2	DNA replication licensing factor MCM2	1
TDP2	Tyrosyl-DNA phosphodiesterase 2	1
REXO4	RNA exonuclease 4	1

Proteins marked in red are DNA binding only
